# Advances and perspectives in animal models of human hepatitis A virus

**DOI:** 10.1002/ame2.70219

**Published:** 2026-05-10

**Authors:** Jian Li, Pei‐Yu Jiang, Hui Zhao, Cheng‐Feng Qin

**Affiliations:** ^1^ State Key Laboratory of Pathogen and Biosecurity Academy of Military Medical Sciences Beijing China; ^2^ School of Basic Medicine Sciences Tsinghua University Beijing China; ^3^ Research Unit of Discovery and Tracing of Natural Focus Diseases Chinese Academy of Medical Sciences Beijing China

**Keywords:** animal models, hepatitis A virus, LNP‐vRNA based mouse model

## Abstract

Hepatitis A virus (HAV) is an important pathogen that has continuously posed a threat to global public health for over 5000 years. The development of accessible and reliable small animal models, especially murine models, is essential for elucidating HAV pathogenesis and advancing preventive and therapeutic strategies. The first mouse model for HAV infection was established using human‐liver chimeric mice, which supported robust viral replication and high viral loads. Later, an *Ifnar1* ^*−/−*^ mice model was established with a specific mouse‐adapted strain, recapitulating key clinical features of human hepatitis A. Recently, to overcome limitations associated with the difficulty in obtaining and amplifying stocks of HAV for animal models, our laboratory established a novel hepatitis A mouse model using lipid nanoparticle‐encapsulated viral genomic RNA (LNP‐vRNA). This approach provides a new strategy for modeling infections of hard‐to‐culture RNA viruses. In this review, we systematically summarize and compare these mouse models, respectively highlighting their advantages and limitations, and offer guidance for their application in future HAV research.

## Graphical abstract

Following HAV infection, humans, non‐human primates, and *Ifnar1* ^*−/−*^ mice develop characteristic manifestations of hepatitis A, including fecal viral shedding, elevated serum ALT levels, and inflammatory cell infiltration in the liver. In contrast, HAV‐infected human liver chimeric mice exhibit fecal viral shedding but do not develop clinical features of hepatitis. To overcome the limited ability of pathogenic HAV strains to replicate efficiently in vitro, two mouse models of HAV infection have been established: the HAV‐2m‐based model and the LNP‐vRNA‐based model. In both models, *Ifnar1* ^*−/−*^ mice display fecal viral shedding, increased serum ALT, and hepatic inflammatory infiltration, recapitulating hepatitis A clinical features. HAV‐2m is an engineered recombinant virus capable of efficient replication in vitro while simultaneously inducing hepatitis A symptoms in *Ifnar1* ^*−/−*^ mice. In the LNP‐vRNA model, hepatitis A is established by delivering in vitro‐transcribed viral genomic RNA into mice using lipid nanoparticles.

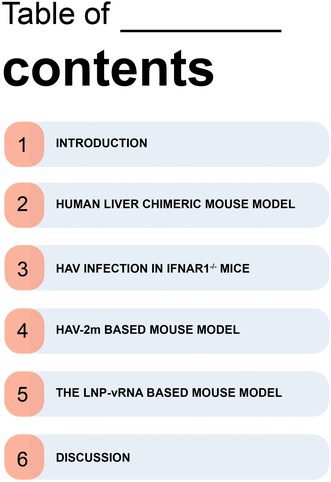

## 1 INTRODUCTION

Hepatitis A virus (HAV) is a positive‐sense, single‐stranded RNA virus belonging to the family *Picornaviridae*. It is transmitted via the fecal‐oral route, with contaminated water and food serving as common sources of infection.^1^ Globally, HAV infects over 100 million people annually, resulting in tens of thousands of deaths.^2^ HAV infection is a self‐recovering disease and currently there are no specific antiviral therapies available for its treatment. Although several vaccines have been developed, which have significantly reduced the incidence of HAV outbreaks and epidemics in economically developed regions, vaccination has not completely eliminated the virus in these areas, as sporadic cases still frequently occur.^3^, ^4^, ^5^ The diagnosis of HAV infection in clinical practice is commonly based on the detection of serum anti‐HAV IgM antibodies, elevated alanine aminotransferase (ALT) levels, and the presence of viral RNA load in stool samples (https://bestpractice.bmj.com/topics/en‐gb/126). Despite the global public health burden of HAV, research into its pathogenesis and the development of effective therapeutics have been severely constrained by several technical bottlenecks. First, HAV infection in cultured cells fails to induce cytopathic effects, making it difficult to assess the pathogenicity of viruses in vitro.^6^ Second, HAV has an exceptionally narrow host range, naturally infecting only humans and non‐human primates, which has historically necessitated the use of costly and ethically constrained non‐human primate models.^7^, ^8^ The lack of a tractable small animal model with an intact immune system has therefore been a major barrier to dissecting the molecular mechanisms of HAV‐induced liver injury and immune responses.^9^

Because HAV has a narrow natural host range, restricted primarily to non‐human primates, including chimpanzees, marmosets, owl monkeys, and rhesus macaques, which have historically been the primary model system for in vivo HAV research.^8^, ^10^, ^11^ These animals can be successfully infected via intravenous or oral inoculation and exhibit acute hepatitis phenotypes resembling those observed in humans, characterized by fecal viral shedding, elevated serum ALT levels, and liver histopathology.^8^, ^10^, ^11^ Chimpanzees were one of the most commonly used experimental animals in early HAV studies. Following experimental infection, chimpanzees shed virus in feces for up to 14 weeks. Viremia is notably less pronounced than fecal shedding and typically concludes around 6 weeks post‐infection. Hepatic viral RNA load does not perfectly correlate with fecal shedding patterns, as viral RNA persists in the liver for a considerable period after its clearance from feces. The liver is the only confirmed site of active HAV replication. However, HAV antigens can be detected in multiple extrahepatic tissues and organs. For instance, besides hepatocytes and Kupffer cells, HAV antigens have been identified in the spleens, kidneys, and lymph nodes of infected primates.^12^, ^13^ While the natural transmission route of HAV is fecal‐oral, the intravenous route is the most commonly employed method for establishing HAV infection in non‐human primates.^14^, ^15^

**FIGURE 1:**
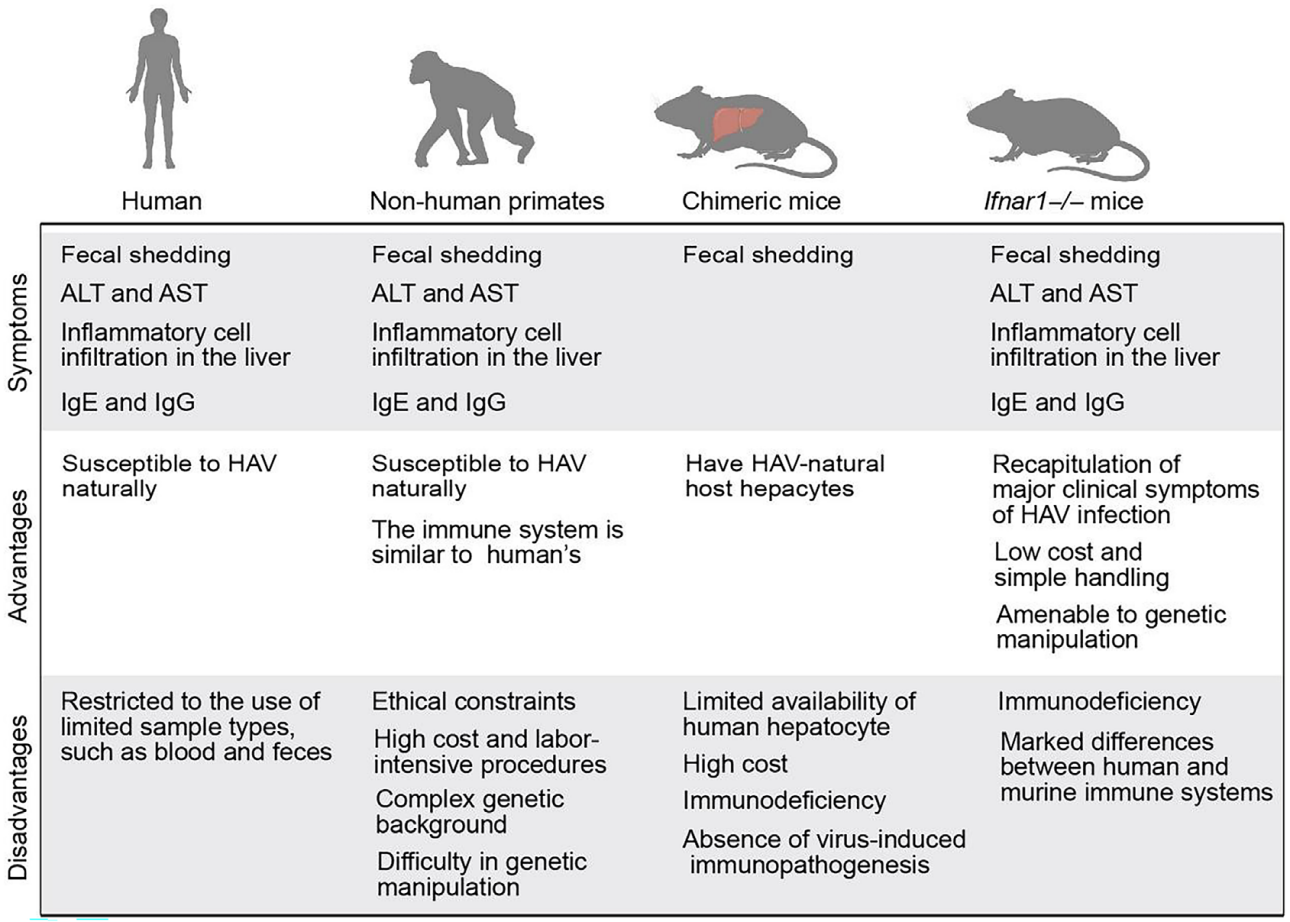
Comparative characterization of current animal models of HAV. The existing HAV animal models encompass non‐human primates, human liver chimeric mice, and *Ifnar1* ^*−/−*^ mice. The figure depicts the symptoms, advantages, and disadvantages of different types of animal models. ALT, alanine aminotransferase; AST, aspartate transaminase; IgE, immunoglobulin E; IgG, immunoglobulin G.

Non‐human primates have long reproductive cycles, and their utilization in experimental research is associated with high costs, technical complexity, strict ethical constraints, complex genetic backgrounds, and limited sample sizes (Figure 1). These inherent limitations have greatly hindered the progress of in vivo studies on HAV. Therefore, the development of a convenient and reliable small‐animal model of HAV infection is particularly important. As the most widely used laboratory animal, mice offer several advantages, including rapid reproduction, low maintenance cost, well‐defined genetic backgrounds, and amenability to genetic manipulation, making them an ideal model for investigating viral infections. Thus, significant efforts have been directed toward establishing murine models of HAV infection. These models—ranging from human‐liver chimeric mice^7^ to immunodeficient *Ifnar1* ^−/−^ mice,^16^ and more recently to a reverse genetics‐enabled HAV‐2m model^17^ and an LNP‐vRNA‐based infection model^18^—have begun to unravel key aspects of HAV biology, including viral entry,^19^ innate immune evasion,^20^ the unexpected role of MAVS‐dependent signaling in hepatocyte apoptosis,^16^, ^21^ and the challenge to the conventional understanding that immune cells such as T, NK cells are the key drivers of hepatitis A pathogenesis.^16^, ^17^, ^22^ In this review, we systematically summarize and compare these emerging mouse models, highlighting their respective advantages and limitations, and provide guidance for their application in future HAV research.

## 2 HUMAN LIVER CHIMERIC MOUSE MODEL

Hepatitis viruses generally exhibit a narrow host range and strong species specificity. Beyond HAV, Hepatitis B Virus (HBV), Hepatitis C Virus (HCV), and Hepatitis E Virus (HEV) also demonstrate a marked resistance to infecting wild‐type mice. In 2001, Dandri et al. successfully used human liver chimeric mice to establish an HBV infection model; in the same year, Mercer et al. also successfully employed chimeric mice for HBV infection studies.^23^, ^24^, ^25^ Human liver chimeric mice have also been primary candidates for developing murine models for HAV.^7^, ^20^, ^26^ In Alb‐uPA/SCID mice inoculated with the HM175 strain, viral genomic RNA became detectable in both serum and feces at the first week post‐inoculation. The viral load in serum and feces peaked around 3 weeks post‐inoculation, reaching levels of 1.6 × 10^6^ GE/mL in serum and 3.4 × 10^9^ GE/g in feces.^7^ Unlike the typical infection kinetics observed in humans or non‐human primates, the HAV RNA load in these mice remained at peak levels until the experimental endpoint at 10 weeks post‐infection. Immunofluorescence analysis of liver sections revealed that viral antigens were predominantly localized within the engrafted human hepatocytes, indicating a selective tropism of HAV for human liver cells. The infectious characteristics of the produced virions were confirmed by inoculating naive humanized mice with serum from previously infected chimeric mice, which resulted in a measurable increase in viral genomic RNA in the serum and feces of the new recipients.^7^ In infected humans and non‐human primates, HAV circulates in the blood as enveloped particles (with a buoyant density of 1.06–1.10 g/cm^3^) and is shed in feces as naked, non‐enveloped virions (1.22–1.28 g/cm^3^).^7^ Consistent with this, serum from infected chimeric mice primarily contained enveloped HAV particles, while feces contained predominantly naked particles.^7^ Despite the natural fecal‐oral transmission route, oral gavage of HAV failed to establish a productive infection in the chimeric mice, as no viral RNA was detected in their serum or feces. This aligns with observations in non‐human primates, where intravenous inoculation proves significantly more efficient than oral administration for establishing infection.^14^, ^15^ The underlying mechanisms responsible for this differential susceptibility based on the route of infection remain an area for further investigation.

Consistent with observations in HAV‐infected non‐human primates,^27^ HAV infection in humanized liver chimeric mice elicited only a limited interferon‐stimulated gene (ISG) response.^7^, ^26^, ^27^ While the infection triggered a significant upregulation of type I (IFN‐α and IFN‐β) and type II (IFN‐γ) interferons in the liver, the expression levels of type III interferons (IL‐28 and IL‐29) and canonical ISGs such as ISG15 and OAS2 remained unchanged. And the expression of several other ISGs, including IFIT2, IRF3, STAT1, IFI27, and MX1, was significantly downregulated.^7^, ^26^ These findings collectively suggest a general suppression of the innate antiviral response, which aligns with the attenuated interferon response documented in HAV‐infected chimpanzees. Comparative histological analysis of liver sections from HAV‐infected and naive chimeric mice revealed no significant differences in the extent of inflammatory cell infiltration or hepatocyte necrosis.^7^, ^26^ The absence of hepatitis in this model is attributed to the lack of a functional adaptive immune system in the SCID background, preventing the immunopathological damage typically associated with clinical hepatitis. Therefore, human liver chimeric mice are well suited for investigating host innate immune responses against HAV. For example, using this model, Colasanti et al. demonstrated that the HAV 3ABC protein cleaves human MAVS less efficiently than the HCV NS3‐4A protease, and thus fails to completely abrogate the host innate immune response.^20^ Additionally, other humanized liver models, such as FRG and TK‐NOG mice,^28^, ^29^ are also available. Compared to the Alb‐uPA/SCID model, they offer advantages like better breeding efficiency and stable human hepatocyte repopulation, serving as valuable alternatives for establishing HAV animal models.

Although humanized liver mouse models have been applied in HAV research, standardized quality control metrics remain essential for ensuring experimental reproducibility. First, the extent of human hepatocyte engraftment should be rigorously assessed.^30^ A human hepatocyte chimerism level ≥ 70% is widely considered the benchmark for successful humanization. Serum human albumin (hAlb) levels should be routinely monitored as a surrogate marker, with levels exceeding 1 mg/mL indicating robust engraftment and functional hepatocyte expansion. Second, functional validation of human‐specific liver activity is required.^30^ This includes the detection of human‐specific drug metabolism, particularly cytochrome P450 enzyme activity, and the presence of human‐specific metabolites, confirming metabolic competence of the graft. Third, for HAV infection studies, standardized virological readouts should be incorporated.^7^, ^20^ These include: (i) quantitative measurement of serum, feces and intrahepatic HAV RNA by real‐time quantitative polymerase chain reaction (RT‐qPCR); (ii) detection of viral antigens in liver tissue using immunohistochemistry or immunofluorescence; and (iii) evaluation of viral replication kinetics over time to confirm sustained infection. In sum, a combined assessment integrating chimerism level, human‐specific biomarkers, metabolic function, and viral replication capacity is recommended to ensure model fidelity and reproducibility across studies.

The HAV‐infected Alb‐uPA/SCID humanized liver mouse model has been proven applicable for antiviral drug evaluation and for investigating the host innate immune response triggered by HAV infection.^7^, ^26^ However, a significant limitation of this model is its failure to recapitulate the hallmark hepatitis pathology observed in natural infections. Furthermore, the high cost, technical complexity, and labor‐intensive process involved in generating and maintaining these chimeric mice substantially constrain the widespread application of this model system.

## 3 HAV INFECTION IN *IFNAR1* ^*−/−*^ MICE

HAV is sensitive to type I interferon, and its replication is potently suppressed by the IFN‐mediated antiviral response.^31^ During HAV infection in primates, the viral 3ABC protein suppresses type I interferon production and facilitates viral establishment by cleaving the host MAVS protein. However, the 3ABC protein of HAV is unable to cleave murine MAVS, thereby preventing efficient infection of wild‐type mice.^9^, ^20^ In 2016, Lemon and colleagues showed that *Ifnar1* ^*−/−*^ C57BL/6 mice were susceptible to HAV infection and developed classical clinical and pathological features of hepatitis A.^16^ Inoculation with the HM175 strain or its progeny viruses in mouse leads to persistent fecal viral shedding for over 3 months, with hepatic viral RNA persisting for more than 160 days in *Ifnar1* ^*−/−*^ mice.^16^ Furthermore, infection in this immunodeficient model recapitulates key pathological features of hepatitis A, including elevated serum ALT levels and inflammatory cell infiltration into the liver parenchyma.^16^ The peak viral load in both feces (approximately 10^6^ GE/mg) and liver (approximately 10^6^ GE/μg RNA), alongside the peak in serum ALT, typically occurs within the first 2 weeks post‐inoculation, followed by a gradual decline.^16^, ^22^

Bulk liver transcriptome and RT‐qPCR analyses revealed a significant upregulation of genes encoding interferons (*Ifnb1, Ifnl2, Ifnl3*), chemokines (*Ccl7*, *Ccl5*, *Cxcl10*), and ISGs (*Ifit1*, *Ifit2*, *Ifit3b*, *Oas3*, *Ifi44*, *Rsad2*, *Isg15*) in *Ifnar1* ^*−/−*^ mice infected with mouse‐adapted HM175‐derived virus. This robust innate immune activation stands in stark contrast to the limited ISG induction observed in HAV‐infected human liver chimeric mice.^16^, ^21^, ^32^ In this mouse model of HAV‐induced liver injury, apoptotic hepatocytes are typically surrounded by immune cells, including NK cells, NKT cells, CD4^+^ T cells, CD8^+^ T cells, and macrophages.^16^, ^17^, ^22^ While these infiltrating immune cells have historically been implicated as the primary drivers of liver damage, studies in *Ifnar1* ^*−/−*^ mice indicate that their depletion or functional impairment does not ameliorate HAV‐induced liver injury.^16^, ^17^, ^22^, ^32^

HAV‐induced liver injury in *Ifnar1* ^−/−^ mice is dependent on the host MAVS‐IRF3 signaling axis. Compared to *Ifnar1* ^−/−^ mice, *Mavs* ^−/‐^ *Ifnar1* ^−/−^ mice infected with HAV exhibit higher viral RNA loads in both feces and liver, yet they do not develop the hepatitis symptoms of elevated serum ALT.^16^ The mechanism whereby the 3ABC protease cleaves MAVS to inhibit IFN production enables HAV to establish infection and cause liver injury in primates. Paradoxically, the absence of liver injury in HAV‐infected *Mavs* ^−/−^ mice indicates that a residual fraction of uncleaved MAVS protein is required for the development of hepatitis. Consistently, it has been reported that HAV 3ABC is a less potent inhibitor of MAVS than the HCV NS3‐4A protease and fails to completely cleave MAVS during infection.^20^ Furthermore, introducing a loss‐of‐function mutation at the IRF3 phosphorylation site in *Ifnar1* ^−/−^ mice (*Irf3* ^S1/S1^ *Ifnar1* ^−/−^) also results in increased hepatic and fecal viral RNA loads post‐HAV infection and significantly attenuates liver injury and hepatitis symptoms,^21^ while HAV infection in *Irf3* ^S1/S1^ *Ifnar1* ^−/−^ mice still induces significant upregulation of genes like *Rsad2*, *Ifit1/2/3*, *Ccl5*, and *Cxcl10*, and the expression of *Ifnl2* and *Ifnl3* (type III interferons) is markedly reduced compared to infected *Ifnar1* ^−/−^ mice.^21^ However, genetic ablation of the interferon‐lambda receptor (*Ifnlr* ^−/−^) in *Ifnar1* ^−/−^ mice does not alleviate HAV‐induced liver injury, indicating that the type III interferon response is not the primary mediator of pathology downstream of phospho‐IRF3.^21^ The specific downstream effectors of IRF3 phosphorylation that govern HAV pathogenicity remain to be fully elucidated. To better align the process of HAV infection in mice with that in primates, a genetically modified mouse strain (*Mavs* ^vs/vs^) was generated. This model carries a modified murine MAVS protein engineered to contain the 3ABC cleavage site.^9^ However, HAV failed to establish a productive infection in the *Mavs* ^vs/vs^ mice and did not induce significant hepatitis pathology.^9^

Rapid, accessible, and reproducible animal disease models for HAV infection are crucial for elucidating its pathogenesis and developing therapeutic strategies. However, a significant bottleneck exists: the HM175 strain and its mouse‐adapted derivatives are notoriously difficult to culture in vitro and are not amenable to efficient reverse genetic manipulation. For instance, transfecting FRhK‐4 cells with in vitro‐transcribed (IVT) RNA from the HM175 strain requires continuous passaging for over 120 days before minimal viral antigen production is detectable.^33^ Consequently, the generation of viral seed stocks necessary for establishing these animal models relies on serial passage within host animals.^16^, ^21^, ^22^ This process is not only time‐consuming and labor‐intensive but also introduces the risk of selecting for adaptive mutations during in vivo passaging, which can compromise the genetic stability and consistency of the resulting animal models.

## 4 HAV‐2m BASED MOUSE MODEL

**FIGURE 2:**
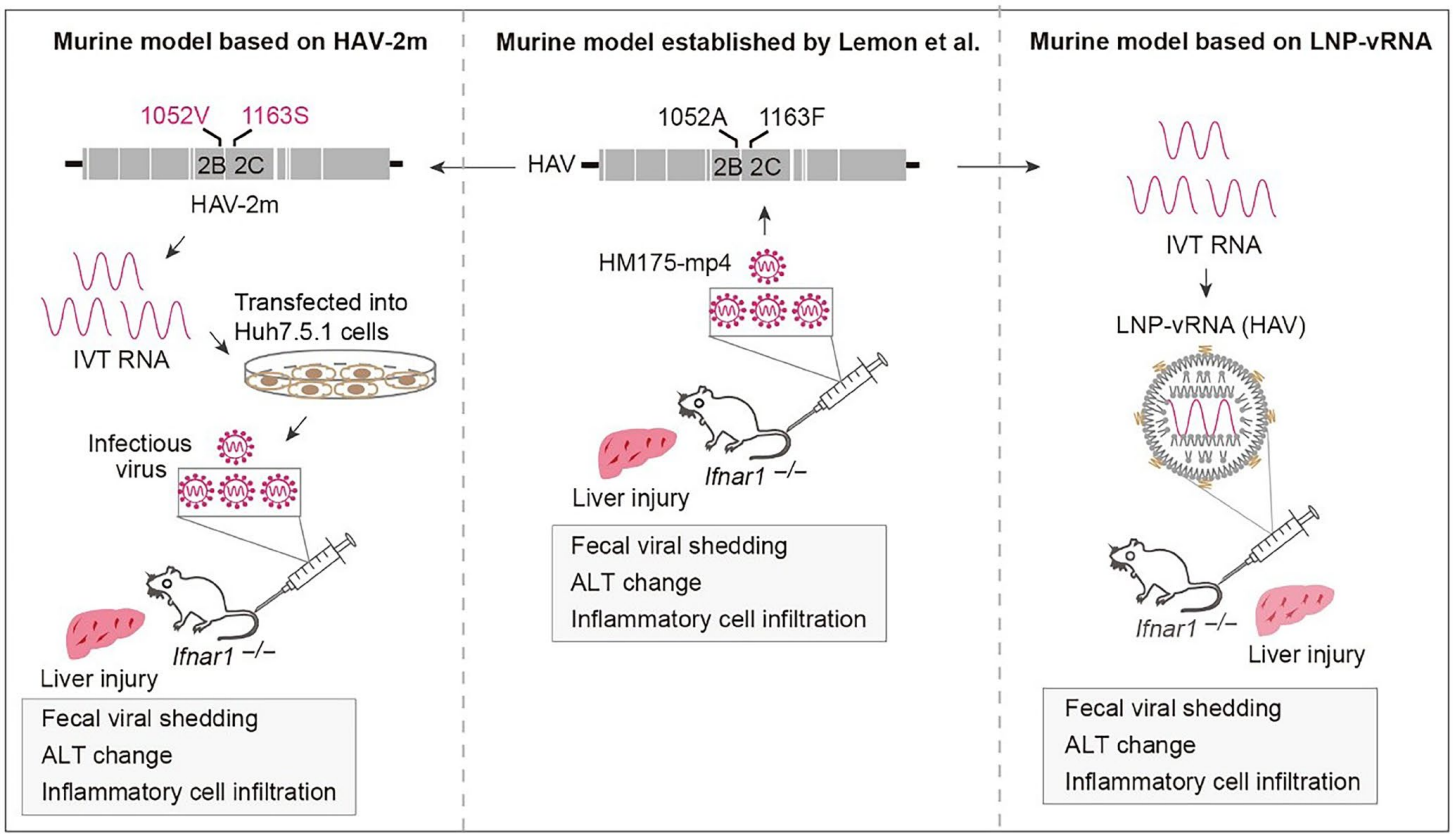
Constructions and characteristics of mouse models based on HAV‐2m and LNP‐vRNA. The middle illustration depicts the HAV infection murine model established by Lemon et al. in *Ifnar1* ^*−/−*^ mice using the mouse adaptation strain HM175‐MP4. Building upon this foundation, we have devised two novel mouse infection models. The left illustration shows the construction of the murine model based on HAV‐2m, while the right illustration presents the construction of the murine model based on LNP‐vRNA. Both of these mouse infection models can manifest fecal viral shedding, elevated alanine aminotransferase levels, along with apoptotic hepatocytes and infiltration of inflammatory cells in the liver. ALT, alanine aminotransferase; IVT RNA, in vitro transcription RNA; LNP‐vRNA, lipid nanoparticle‐encapsulated viral genomic RNA.

The A1052V and F1163S substitutions represent the most critical and frequently observed adaptive mutations during the in vitro culture adaptation of HAV. Introducing these two mutations into the wild‐type HM175 strain significantly enhances viral replication efficiency in cell culture.^34^ To establish a more convenient murine model for HAV infection, our laboratory simultaneously introduced these mutations into the well‐characterized HM175‐mp4 strain, generating an engineered recombinant virus designated HAV‐2m.^17^ HAV‐2m overcomes the challenge of inefficient in vitro proliferation of HAV wild‐type strains, allowing for highly efficient propagation and facilitating reverse genetic manipulations (Figure 2). Transfection of Huh7.5.1 cells with IVT RNA of HAV‐2m, followed by serial passaging, yields infectious HAV‐2m virus. Infection of Huh7.5.1 cells with this virus results in viral RNA titers reaching up to 2 × 10^8^ GE/mL at 6 dpi. In contrast, transfection with IVT RNA from HM175‐mp4 followed by serial passaging does not yield detectable levels of viral genomic RNA in Huh7.5.1 cells. The HAV‐2m genome demonstrates relative stability, with no reversion of the A1052V and F1163S mutations observed after four serial passages.

Intravenous inoculation of *Ifnar1* ^−/−^ mice with 1 × 10^8^ GE of HAV‐2m leads to persistent viral shedding in feces until the mice are sacrificed at 28 dpi.^17^ Serum ALT levels are significantly elevated at 7 dpi and peak at 14 dpi. Histopathological examination of liver sections collected at 14 dpi via H&E staining reveals substantial hepatocyte apoptosis and inflammatory cell infiltration within the liver parenchyma. Single‐cell RNA sequencing and flow cytometric analysis of livers from HAV‐2m‐infected mice at 7 dpi show that the immune cells infiltrating the liver include T cells, NK cells, monocytes, Neu_Isg15 cells, dendritic cells, and macrophages, accounting for 45.97%, 14.18%, 7.67%, 7.17%, 3.86%, and 3.29% of the captured cells, respectively. Compared to naive mice, the proportions of these immune subsets increased by 3.93‐fold, 4.82‐fold, 4.06‐fold, 2.22‐fold, 2.31‐fold, and 6.54‐fold, respectively. However, simultaneous depletion of these immune cells in *Ifnar1* ^−/−^ mice using clodronate liposomes and a cocktail of depleting antibodies targeting macrophages, T cells, NK cells, neutrophils, and monocytes did not ameliorate the HAV‐induced elevation in serum ALT levels or hepatocyte apoptosis. This evidence suggests that HAV‐triggered hepatocyte apoptosis may occur primarily through an “intrinsic” apoptosis pathway that is independent of these immune effector cells.

Leveraging the ease of reverse genetics with HAV‐2m, this model was utilized to evaluate the impact of the key adaptive mutation K1118M on HAV pathogenesis. Following intravenous inoculation of *Ifnar1* ^*−/−*^ mice with an identical dose of HAV‐2m and HAV‐2m_K1118M, the HAV‐2m_K1118M mutant failed to induce the significant serum ALT elevation and hepatic inflammatory cell infiltration that were observed in HAV‐2m‐infected mice.^17^ Furthermore, the viral RNA load in the liver of HAV‐2m_K1118M‐infected mice was also lower than that in HAV‐2m‐infected mice at 14 dpi.^17^ The results demonstrate that while the K1118M substitution enhances the replication capacity of HAV‐2m in vitro, it attenuates viral replication and reduces pathogenicity in infected *Ifnar1* ^−/−^ mice.^17^

## 5 THE LNP‐vRNA BASED MOUSE MODEL

To circumvent the challenges associated with the production of viral stocks for HAV murine models,^16^, ^21^, ^22^ our lab also employed LNPs to deliver the full‐length genomic RNA of the HM175‐mp4 strain directly to the mouse liver, thereby establishing a novel LNP‐vRNA‐based HAV infection murine model.^35^ The relatively long genomic HAV RNA (~7.5 kb) was efficiently encapsulated by LNPs with an encapsulation efficiency of approximately 90%. The resulting LNP‐vRNA particles exhibited a mean diameter of 74.82 nm and a low polymer dispersity index (PDI) of <0.1, indicating a homogeneous preparation.^35^ Experimental data demonstrate that *Ifnar1* ^*−/−*^ mice intravenously inoculated with LNP‐encapsulated HM175‐mp4 genomic RNA (LNP‐mp4) exhibited persistent fecal viral shedding for over 28 days, with peak viral RNA loads reaching 10^7^ GE/g of feces.^35^ Through LNP‐vRNA strategies, including the application of lower doses (with 1 ~ 10 μg being effective) to prevent excessive immune activation and the consequent over‐elimination of dsRNA, we successfully established a stable and pathogenic HAV murine model (Figure 2).

Furthermore, the LNP platform has demonstrated utility beyond HAV, proving equally effective for the delivery of HEV genomic RNA and the establishment of corresponding animal models. Rabbits inoculated with LNPs encapsulating the genomic RNA of the HEV R14 strain (GenBank accession no. OR224867), designated LNP‐R14, exhibited persistent fecal viral shedding for over 180 days and tested positive for HEV antigen in their urine.^35^ This LNP‐vRNA‐based platform for establishing viral infection models offers significant advantages. It greatly facilitates reverse genetics studies, enabling the investigation of key viral virulence determinants and the evaluation of antiviral therapeutics. This approach thus presents a novel and versatile strategy for generating animal models for RNA viruses that are notoriously difficult to culture in vitro. Although the LNP‐vRNA can successfully achieve high‐titer infection and recapitulate the pathogenic HAV phenotype in mice, its delivery method bypasses the natural process of receptor‐mediated viral entry. This inherent limitation restricts the model's fidelity in recapitulating the mechanisms of viral invasion and the dynamics of gradual viral dissemination. Furthermore, the sustained expression of exogenous RNA may non‐specifically activate innate immune pathways, thereby confounding the interpretation of the authentic viral pathogenic mechanisms.

## 6 DISCUSSION

Convenient and reliable small animal models are crucial for disease research. The natural host range of HAV is strictly limited to primates, which historically compelled researchers to rely on non‐human primates for HAV‐related studies. This significantly hindered progress in understanding the virus and its pathogenesis.^8^, ^15^, ^36^ The establishment of the HAV‐infected *Ifnar1* ^−/−^ mouse model resolved this long‐standing challenge in the field. This model recapitulates main clinical features of hepatitis A, including fecal viral shedding, elevated serum ALT levels, and hepatic inflammatory cell infiltration.^16^ Subsequently developed models based on the HAV‐2m and LNP‐vRNA also recapitulate these clinical symptoms.^17^, ^35^ Furthermore, they overcome the major obstacles of inefficient in vitro cultivation of pathogenic HAV strains and the difficulty of performing reverse genetics, thereby greatly facilitating the application of HAV mouse models.^17^, ^35^ These models have substantially advanced our understanding of HAV virology, immunology, and drug development.^16^, ^19^, ^21^, ^22^, ^32^ In virology, for instance, HAVCR1—considered as the HAV receptor—has been shown to be non‐essential for viral entry, as HAV can infect *Ifnar1* ^−/−^ mice lacking HAVCR1.^19^ As for HAV immunology, studies in mice have demonstrated that HAV‐induced liver injury is dependent on the MAVS‐IRF3 signaling axis, whereas depletion of NK cells, T cells, or macrophages does not alleviate HAV‐associated liver damage.^16^, ^21^, ^22^ With regard to HAV antiviral drugs, Lemon et al. demonstrated that the ZCCHC14/TENT4 complex is essential for HAV RNA synthesis. Administration of RG7834 to inhibit the function of the ZCCHC14/TENT4 complex effectively blocked HAV infection in *Ifnar1* ^−/−^ mice and alleviated hepatitis symptoms.^37^

The viral load and pathogenicity of the HAV‐2m recombinant virus in mice are slightly lower than those of the wild‐type HM175 strain and its mouse‐adapted derivatives. Inoculation with 2.6 × 10^8^ GE of HM175‐mp4 virus resulted in hepatic viral RNA loads of approximately 10^6^ GE/μg total liver RNA and peak serum ALT levels around 400 U/L.^16^ In contrast, infection with 2 × 10^8^ GE of HAV‐2m led to hepatic viral loads of 10^5^ GE/μg RNA and a peak ALT of about 200 U/L.^17^ Therefore, despite HAV‐2m exhibiting enhanced replication in cultured cells due to the A1052V and F1163S mutations, it shows reduced viral load and attenuated hepatitis in mice.^16^, ^17^ Nonetheless, HAV‐2m remains an ideal strain for establishing a murine hepatitis A model, as it still causes significant hepatitis symptoms and recruits a similar repertoire of infiltrating immune cells as HM175‐mp4 infection.^16^, ^17^ And the moderate attenuation of HAV‐2m in vivo is not entirely disadvantageous, as it alleviates biosafety concerns associated with gain‐of‐function genetic engineering. The LNP‐vRNA‐based HAV infection mouse model can effectively recapitulate the clinical manifestations of hepatitis A, but its delivery method bypasses the natural process of receptor‐mediated viral entry. And the sustained expression of exogenous RNA may non‐specifically activate innate immune pathways, thereby confounding the interpretation of the authentic viral pathogenic mechanisms. Overall, these small animal models each present distinct advantages and limitations. Compared to LNP‐vRNA‐based HAV infection models, the HM175‐mp4 and HAV‐2m infection models more closely recapitulate the natural course of viral infection and are therefore better suited for studies on HAV virology, immunology, drug development, and vaccine evaluation. In contrast, the LNP‐vRNA‐based infection model has convenience for reverse genetic manipulation of the viral genome, making it particularly useful for investigating functional mutations and identifying key virulence determinants of HAV. Given that the HAV 3ABC protease is unable to cleave murine MAVS,^9^ humanized liver mouse models are therefore suitable for investigating HAV‐mediated interference with the MAVS–IRF3 signaling axis.

The HAV‐infected *Ifnar1* ^−/−^ mouse model, which faithfully recapitulates major human hepatitis A symptoms,^16^ has been proven to be a powerful tool for investigating HAV pathogenesis and developing countermeasures. Utilizing this model, the MAVS‐IRF3 signaling axis was identified as a key driver of HAV‐induced liver injury,^16^, ^21^ while immune cells previously thought to be central, such as T cells, NK cells, and macrophages, were shown to be dispensable for the pathology.^16^, ^22^, ^32^ However, a critical interspecies difference exists. During infection in primates, HAV partially suppresses the MAVS‐IRF3 axis through cleavage of MAVS by its 3ABC protease.^9^, ^16^, ^31^ This fine‐tuning of MAVS‐IRF3 inhibition by 3ABC is therefore a critical determinant of HAV fitness and pathogenicity. Since HAV 3ABC protein does not cleave murine MAVS,^9^, ^16^ HAV cannot use this mechanism to modulate its pathogenicity in *Ifnar1* ^−/−^ mice, suggesting a potential divergence in pathogenic mechanisms between primates and this mouse model. A humanized *Mavs* ^vs/vs^ mice was engineered to address this discrepancy.^9^ Despite the humanized MAVS protein being cleavable by 3ABC, HAV failed to establish a robust infection in *Mavs* ^vs/vs^ mice.^9^ Given that HAV infects *Mavs* ^−/−^ mice efficiently,^16^ the likely explanation is that the cleavage efficiency of 3ABC for the humanized MAVS protein in vivo was insufficient. Future studies may further optimize existing animal models from two perspectives. First, the establishment of immunocompetent mouse models for HAV infection remains a promising direction; enhancing the ability of the HAV 3ABC protease to cleave the MAVS protein through additional mutations could be considered as one potential strategy. Second, it is necessary to develop mouse infection models using other clinical strains. Currently, the viral strains used to establish animal models are primarily derived from the HM175 strain isolated many years ago,^16^, ^21^, ^22^ which belongs to genotype IB, whereas genotype IA is more prevalent in clinical settings. Therefore, it is important to establish hepatitis A mouse models based on genotype IA strains.

Animal models are essential for bridging basic virology and clinical application. A key priority is to use these models to unravel the mechanisms driving the clinical heterogeneity of HAV infection—from asymptomatic cases in children to acute liver failure in adults—by manipulating host factors such as age, genetic background, and pre‐existing liver conditions to identify determinants of disease severity. This approach also supports biomarker discovery and validation, enabling the identification of host factors and viral variants linked to disease progression that can be tested in clinical samples to advance diagnostic and prognostic tools. Concurrently, standardizing humanized liver mouse models will be critical for their broader application in drug development and regulatory toxicology, with unified protocols for model generation, quality control, and outcome assessment enhancing reproducibility and accelerating translation from bench to bedside.

## AUTHOR CONTRIBUTIONS

**Jian Li:** Writing – original draft; writing – review and editing. **Pei‐Yu Jiang:** Writing – review and editing. **Hui Zhao:** Writing – review and editing. **Cheng‐Feng Qin:** Funding acquisition; supervision; writing – review and editing.

## FUNDING INFORMATION

This work was supported by the Foundation of State Key Laboratory of Pathogen and Biosecurity of China (SKLPBS2405). Research in C.F.Q's lab was supported by the National Science Fund for Distinguished Young Scholar (81925025) and the Innovation Fund for Medical Sciences (2019‐I2M‐5‐049) from the Chinese Academy of Medical Sciences.

## CONFLICT OF INTEREST STATEMENT

Chengfeng Qin is an editorial board member of Animal Models and Experimental Medicine (*AMEM*) and a corresponding author of this article. To minimize bias, she was excluded from all editorial decision making related to the acceptance of this article for publication.

## ETHICS STATEMENT

Not applicable.
